# Identification of potential biomarkers and candidate small molecule drugs in glioblastoma

**DOI:** 10.1186/s12935-020-01515-1

**Published:** 2020-08-28

**Authors:** Wei-cheng Lu, Hui Xie, Ce Yuan, Jin-jiang Li, Zhao-yang Li, An-hua Wu

**Affiliations:** 1 Department of Neurosurgery, First Affiliated Hospital of China Medical University, Shenyang, Liaoning China; 2 Department of Histology and Embryology, College of Basic Medicine, Shenyang Medical College, Shenyang, Liaoning China; 3 Graduate Program in Bioinformatics and Computational Biology, University of Minnesota, Minneapolis, USA; 4 Department of Neurosurgery, General Hospital of Northern Theater Command, Shenyang, Liaoning China; 5 Department of Laboratory Animal Center, China Medical University, Shenyang, Liaoning China

**Keywords:** Glioblastoma, Differentially expressed genes, Hub genes, Prognosis, Small molecular drugs

## Abstract

**Background and aims:**

Glioblastoma (GBM) is a common and aggressive primary brain tumor, and the prognosis for GBM patients remains poor. This study aimed to identify the key genes associated with the development of GBM and provide new diagnostic and therapies for GBM.

**Methods:**

Three microarray datasets (GSE111260, GSE103227, and GSE104267) were selected from Gene Expression Omnibus (GEO) database for integrated analysis. The differential expressed genes (DEGs) between GBM and normal tissues were identified. Then, prognosis-related DEGs were screened by survival analysis, followed by functional enrichment analysis. The protein–protein interaction (PPI) network was constructed to explore the hub genes associated with GBM. The mRNA and protein expression levels of hub genes were respectively validated in silico using The Cancer Genome Atlas (TCGA) and Human Protein Atlas (HPA) databases. Subsequently, the small molecule drugs of GBM were predicted by using Connectivity Map (CMAP) database.

**Results:**

A total of 78 prognosis-related DEGs were identified, of which10 hub genes with higher degree were obtained by PPI analysis. The mRNA expression and protein expression levels of *CETN2*, *MKI67*, *ARL13B*, and *SETDB1* were overexpressed in GBM tissues, while the expression levels of *CALN1*, *ELAVL3*, *ADCY3*, *SYN2*, *SLC12A5*, and *SOD1* were down-regulated in GBM tissues. Additionally, these genes were significantly associated with the prognosis of GBM. We eventually predicted the 10 most vital small molecule drugs, which potentially imitate or reverse GBM carcinogenic status. Cycloserine and 11-deoxy-16,16-dimethylprostaglandin E2 might be considered as potential therapeutic drugs of GBM.

**Conclusions:**

Our study provided 10 key genes for diagnosis, prognosis, and therapy for GBM. These findings might contribute to a better comprehension of molecular mechanisms of GBM development, and provide new perspective for further GBM research. However, specific regulatory mechanism of these genes needed further elaboration.

## Background

Glioblastoma (GBM) is a most common and aggressive malignant brain tumor, accounting for 16% of all primary brain and central nervous system neoplasms [1]. The mean survival of GBM is approximately 14.6 months, and GBM is one of the most challenging malignancies to treat due to its high heterogeneity, high recurrence rate, and diffusing invasiveness [2]. Despite extensive efforts to explore novel therapies, the survival of GBM has not markedly improved. Therefore, it is necessary to develop effective treatment options. Currently, gene therapy, molecularly targeted therapy, and immunotherapy are promising treatment approaches [3].

Extensive studies have reported the biomarkers and drug targets for GBM treatment. Previous study indicated that genes such as estrogen receptor 2 (*ESR2*), ELOVL fatty acid elongase 6 (*ELOVL6*), iroquois homeobox 3 (*IRX3*), PDZ binding kinase (*PBK*), centromere protein A (*CENPA*), and kinesin family member 15 (*KIF15*) were significantly associated with the prognosis of GBM, suggesting that these genes might be potential targets for GBM treatment [4, 5]. Additionally, drugs like triple-drug therapy (bevacizumab, irinotecan, and temozolomide) had benefit effect on recurrent GBM [6]. However, different studies often yield diverse results and the molecular mechanism of GBM pathogenesis has not been entirely elucidated. Thus, it is desperately required to explore novel biomarkers and small drug molecules.

Currently, the microarray gene expression research has been performed to uncover the molecular mechanism of various cancers. The mRNA data are collected from two databases, including Gene Expression Omnibus (GEO) and The Cancer Genome Atlas (TCGA). The GEO database can be applied to identify the differentially expressed genes (DEGs), explore molecular signal and its correlation, and analyze gene regulation network [7]. However, due to the limited samples, the analysis results of a single microarray dataset may be biased and unreliable. Hence, integrated analysis of multiple datasets can improve the accuracy and reliability of the results, thus obtain a comprehensive discovery of DEGs in tumors.

**Fig. 1:**
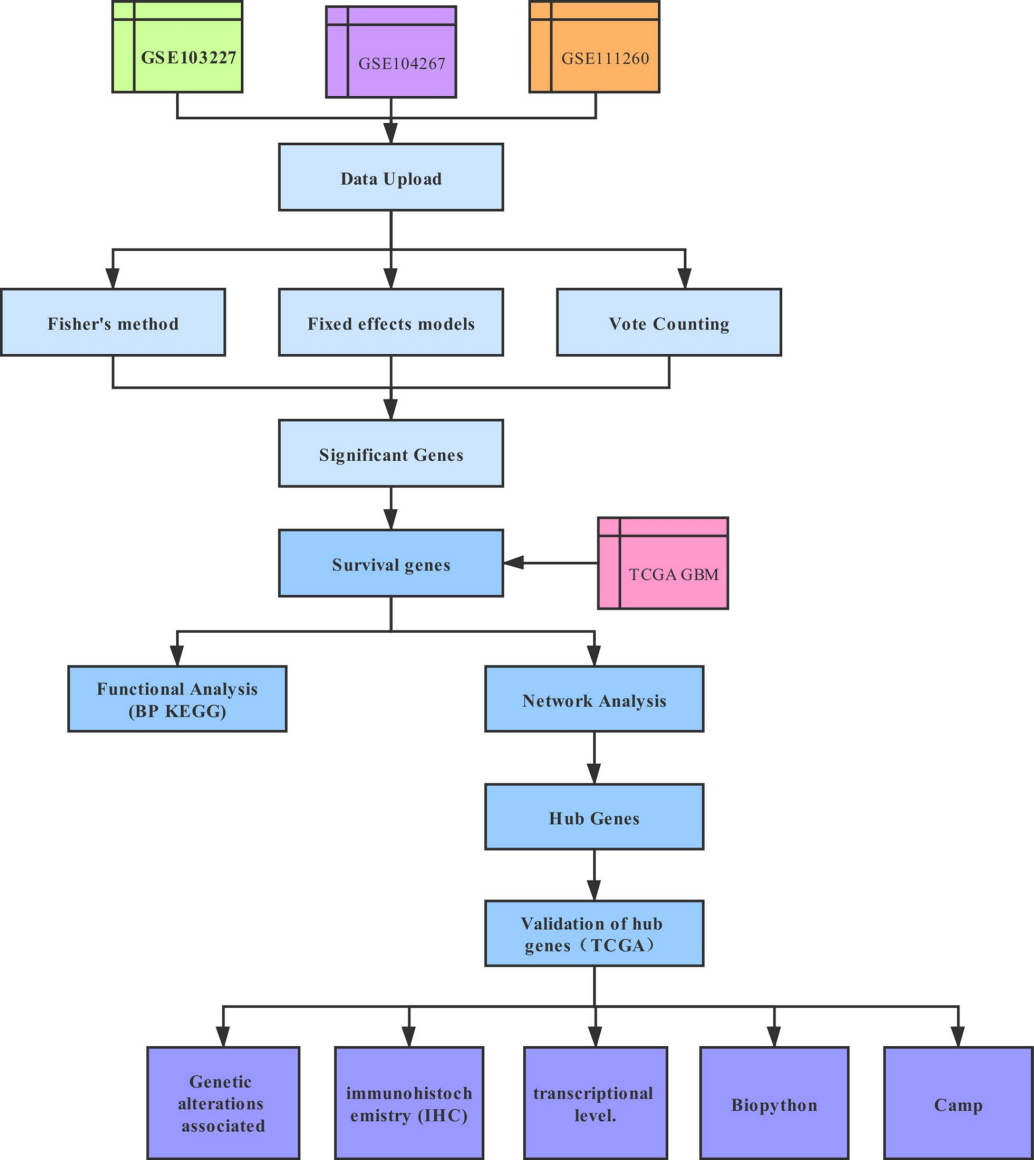
A flow chart of this study protocol

In the present study, three microarray datasets related GBM were selected for further study, the raw data of mRNA profile were downloaded from GEO database and integrated bioinformatics analyses of three data sets were conducted. The overlapping DEGs were identified by the intersection of three datasets. Then, the DEGs associated with GBM prognosis were screened using TCGA database. Functional enrichment analysis was performed to understand the biological functions of these DEGs. We also established a protein–protein interaction (PPI) network to screen hub genes. Thereafter, the mRNA and protein expression level of hub genes were respectively verified by using UALCAN online tool and Human Protein Atlas (HPA) database. Finally, the small molecule drugs of GBM were explored by connectivity map (CMAP) database. The flow chart of this study protocol is shown in Fig. 1.

## Methods and materials

### Microarray data

GBM datasets were retrieved from the GEO database (http://www.ncbi.nlm.nih.gov/geo) [8] using the keywords “microarray & GBM”. The limitation criteria included: (1) date of publication from 2017 to 2019; (2) tissue samples gathered from human GBM and normal tissues; (3) studies included at least 10 samples; and (4) the samples were not treated by any chemical or physical treatment. Finally, three datasets (GSE111260, GSE103227, and GSE104267) met our criteria, and the detailed information is listed in Table 1.

**Table 1:** Characteristics of studies composing the gene expression compendium

Dataset	Study(Citation)	Platform	Organism	Sample (Glioblastoma)	Sample (Control)
GSE103227	Chun luo, 2018	Agilent-045997 Arraystar human lncRNA microarray V3	Homo sapiens	5	5
GSE104267	Jianjun Gu 2017	Phalanx Human lncRNA OneArray v1_mRNA	Homo sapiens	9	3
GSE111260	Jeanmougin Jeanmougin, 2018	Affymetrix Human Exon 1.0 ST Array	Homo sapiens	67	3

### Data pre-processing

The preprocessing of raw data was performed using the limma package of the R software (Version 3.34.9, http://www.bioconductor.org/packages/release/bioc/html/limma.html) [9], and then data normalization of samples from each expression profiles was conducted by using robust multi-array average (RMA) method [10, 11], including background adjustment, quantile normalization, and log 2 conversion. Afterwards, the probes were annotated with the platform annotation file. The probes that did not matched the gene (gene symbol) were removed; in addition, for the multiple probes that mapped to the same gene, their average values were calculated as the final expression value.

### DEGs screening and Meta-analysis

DEGs between GBM and control sample in the three datasets were respectively screened by using the Limma package. P < 0.05 and |log Fold change (FC)| > 1 were considered as the criteria for DEGs.

The integration of DEGs from three microarray datasets was conducted by Network Analyst 3.0 database [12] (https://www.networkanalyst.ca/NetworkAnalyst/uploads/MetaLoadView.xhtml), which could compare and analyze of DEGs generated from different studies via various statistical methods. In this study, three statistical methods, including Fisher’s method, Fixed effects models, and Vote Counting, were applied to integrate multiple data sets. DEGs with P < 0.001 (both Fisher’s method and Fixed effects models) and vote counts ≥ 2 were considered as shared DEGs. Meanwhile, the ComBat function of the R package sva [12] was utilized to eliminate heterogeneity between these three datasets.

### VENN analysis

The DEGs obtained from each dataset were analyzed by VENN analysis to observe the up- or down-regulation of the genes. Additionally, the DEGs screened by the three integration methods were also analyzed by VENN, and the DEGs that existed in at least two methods were selected as the focus of the further analysis.

### Survival analysis

Both the mRNA-seq data and clinical information of GBM patients were acquired from TCGA genomic data commons (GDC) (https://xenabrowser.net/) portal [13]. According to the shared DEGs identified from the integrated analysis, the samples with no overall survival (OS) time (or less than one mouth) and the DEGs with median expression level less than 0 were removed. Afterwards, the remaining samples were divided into high expression group and low expression group based on the median expression levels of genes. Survival analysis was performed using Kaplan-Meier and the log-rank statistical test. P < 0.05 was regarded as statistically significant threshold.

### Functional enrichment analysis of prognostic related DEGs

To investigate the biological functions and pathways involved in these prognostic related DEGs, the Gene Ontology (GO) terms and pathway analysis were performed by using metascape database (http://metascape.org) [14]. Metascape utilized hypergeometric test and Benjamini–Hochberg *P* value correction algorithm to identify all statistically enriched terms (GO or KEGG terms). P < 0.01 and count > 3 were set as the threshold of significantly enriched terms. In order to further explore the relationship between the significantly enriched terms, the kappa-statistical similarities of these terms were calculated, and the overlapping or related terms were identified to perform functional network clustering. According to the gene similarity enriched in each term (similarity of > 0.3), the interaction relationship of the terms was obtained. Subsequently, the functional enrichment network was constructed.

### The PPI network construction

The prognostic related DEGs were mapped into Search Tool for the Retrieval of Interacting Genes (STRING, version: 11.0, https://string-db.org) database [15] to recognize their potential interaction relationships from protein level. The species was *Homo sapiens* and the confident interaction score more than 0.15 (low confident) was set as significant interaction. The PPI network was visualized using Cytoscape software (version: 3.6.1, http://www.cytoscape.org/) [16]. In addition, the degree of each protein node was calculated and nodes with degree ≥ 10 were selected as hub genes.

### Verification of hub genes

We used the online software UALCAN (http://ualcan.path.uab.edu/index.html) [17] to verify the hub genes identified from the PPI network. The candidate hub genes were submitted to the UALCAN database and the TCGA data were applied to validate the relationship between the genes expression and the prognosis of GBM.

### Gene mutation analysis

The cBio Cancer Genomics Portal could analyze the molecular data obtained from cancer tissues and cytology, to recognize and understand the heredity, epigenetics, and gene expression. Thus, we used the CBiocancer genomics portal (https://www.cbioportal.org/) [18] to analyze the genetic mutations of the key genes among samples.

### Immunohistochemical analysis

The HPAs database, composed of tissue atlas, cell atlas, and pathology atlas, is provided the data of transcriptomics and proteomics in specific human tissues. In this study, the protein level of hub genes in GBM tissues and compared normal tissues was investigated by using HPA database [19].

### Identification of candidate small molecule drugs for GBM

The CMAP database, composed of 7056 gene expression profiles induced by 1309 small molecules, is widely applied to explore the potential unknown roles of existing drugs on diseases [20]. First, the prognosis related genes were classified into up-regulated and down-regulated groups. Then, these genes from two groups were uploaded into CMAP database to obtain the potential small drug molecules, and P < 0.05 was regarded as the cut-off criteria. Finally, the enrichment scores (− 1 to + 1) that could assess the similarity between genes and drugs were calculated. Specifically, enrichment score > 0 indicated the molecules had potential synergistic effects to GBM, suggesting they were able to imitate the biological status of GBM cell; while enrichment score < 0 revealed molecules had potential antagonistic effects, indicating they could reverse the GBM carcinogenic status and could serves as therapeutic drugs.

## Results

### Identification of DEGs from GEO datasets analysis

**Fig. 2:**
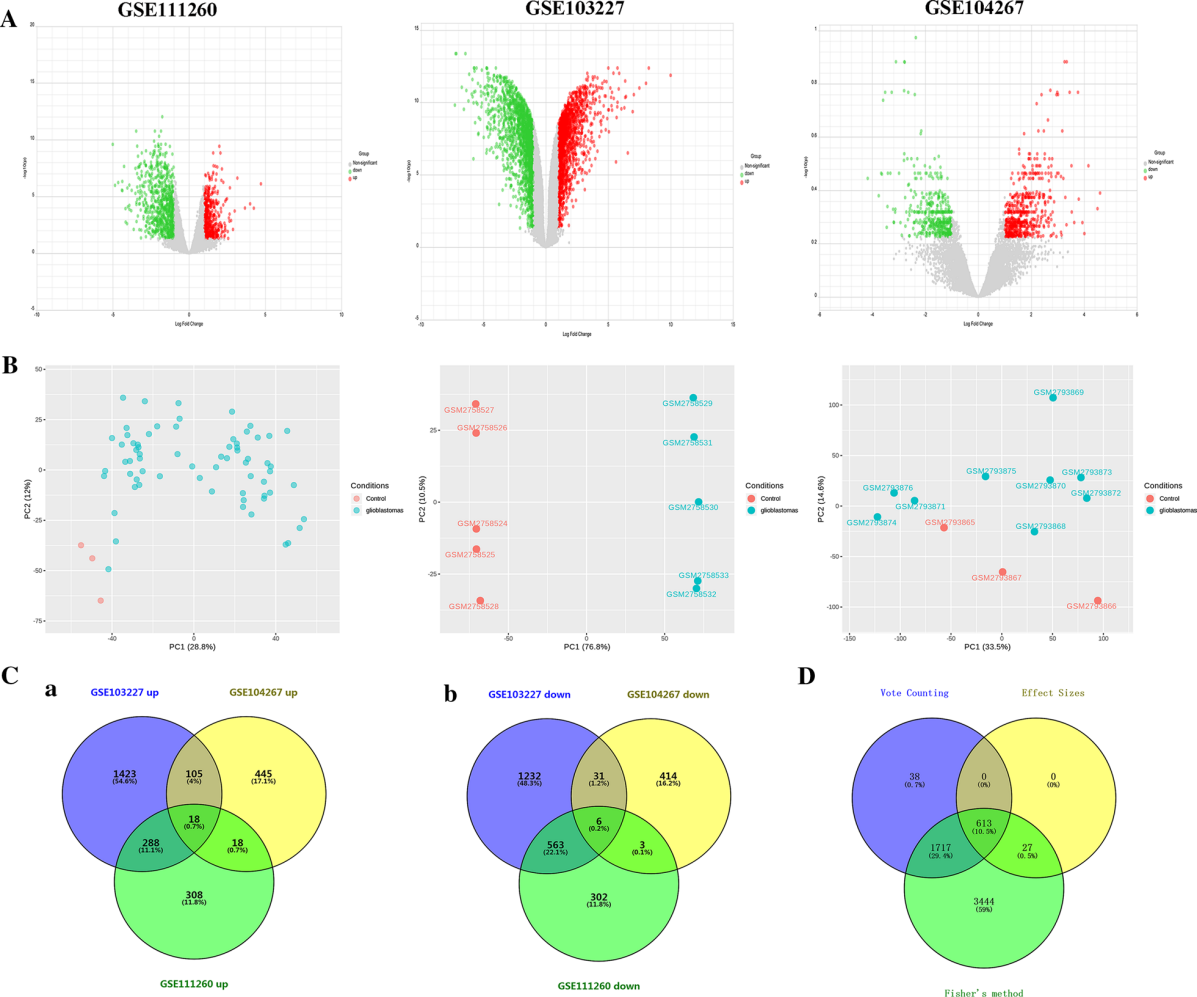
Identification of DEGs in three GEO datasets. **A:** Volcano plot. Green indicates down-regulated DEGs, and red indicates up-regulated DEGs. **B**: PCA plot. Red represents control sample, and blue represents GBM sample. **C**: VENN diagram of DEGs identified from three datasets (a: up-regulated DEGs, b: down-regulated DEGs). **D**: The DEGs identified by three statistical methods

The raw data from three gene expression profiles (GSE103227, GSE104267, and GSE111260) were downloaded from NCBI GEO database. There were 81 GBM samples and 11 normal samples in this study. DEGs between GBM samples and normal samples were screened from three studies, and then visualized by volcano plots and Principal Components Analysis (PCA) score plots (Fig. 2A, B). Afterwards, the number of DEGs obtained from three datasets is shown in Additional file 1: Table S1. Furthermore, the Venn diagrams showed that 24 overlapping DEGs were obtained among three datasets, including 18 up-regulated genes (Fig. 2Ca) and 6 down-regulated genes (Fig. 2Cb).

### Meta-analysis of three GEO datasets

By employing three statistical methods, a total of 5801, 640, and 2368 DEGs were identified by Fisher’s method, Fixed effects models, and Vote counting, respectively. Additionally, 613 shared genes were obtained by all three statistical methods and 2357 DEGs were existed in at least two methods (Fig. 2D).

### Survival analysis of DEGs

In order to clarify the relationship between gene expression and GBM prognosis, we used K-M and log rank test for survival analysis. The clinical data of 167 patients with GBM was downloaded from TCGA, and the overall survival analysis of 2357 DEGs was performed. Finally, we obtained 78 DEGs were significantly connected with the prognosis of GBM (Additional file 2: Table S2).

### GO enrichment and KEGG pathway analysis of prognosis related genes

**Fig. 3:**
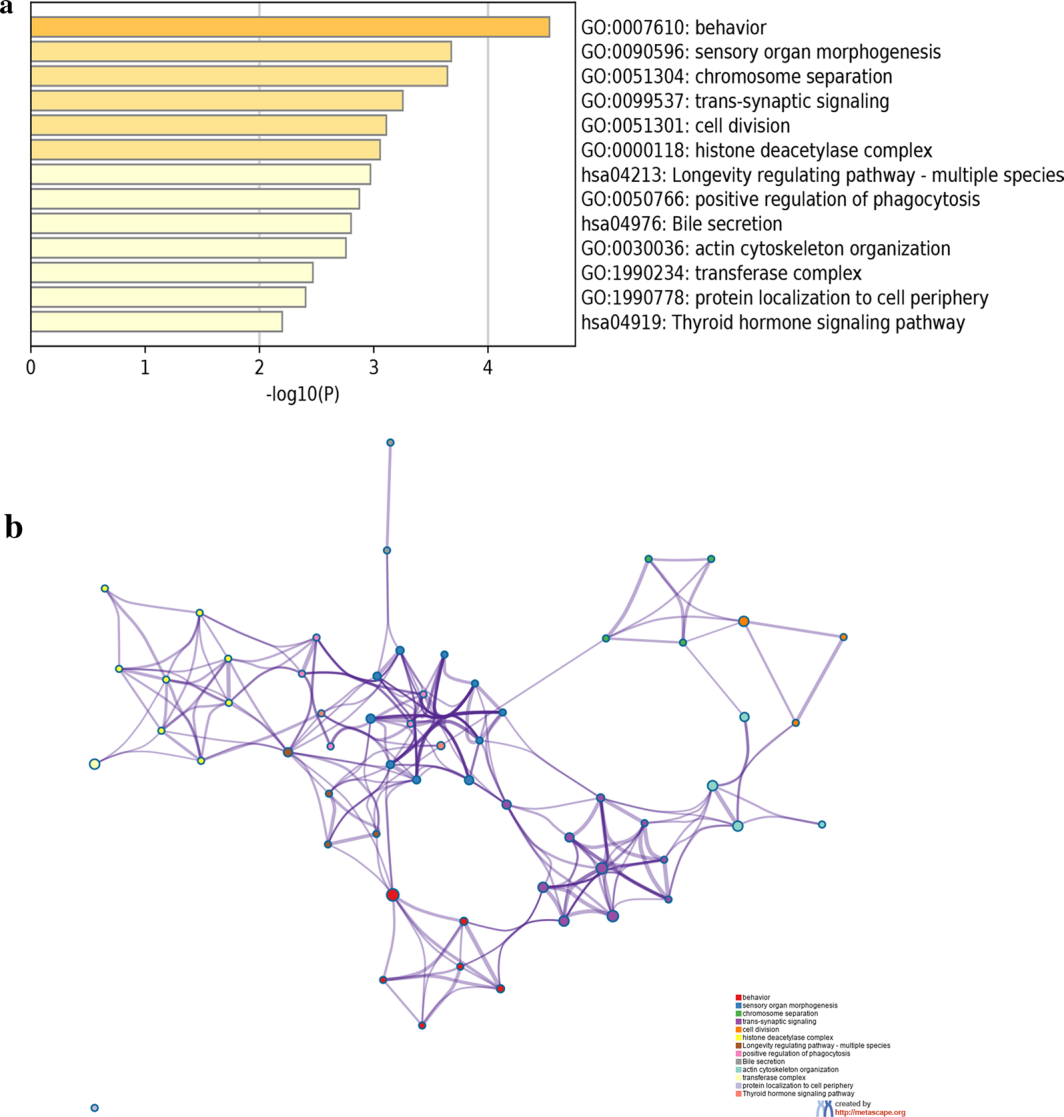
Functional enrichment analysis of prognosis-related DEGs. **a**: Top 13 clusters from Metascape pathway enrichment analysis of prognosis-related DEGs. **b**: Network of GO and KEGG enriched terms colored by clusters. Nodes of the same color belong to the same cluster. Terms with Kappa similarity score > 0.3 are linked by an edge

According to the result mentioned above, the functional enrichment analysis of 78 prognosis-related genes was conducted. Three categories of GO enrichment analysis were performed, including biological process (BP), cellular component (CC), and molecular function (MF). The results indicated that these genes were mainly associated with GO_BP terms such as behavior, sensory organ morphogenesis, and chromosome separation. As for GO_CC terms, genes were primarily enriched in histone deacetylase complex, neuron to neuron synapse, transferase complex, and axon. For MFs, DEGs were particularly related to DNA-binding transcription repressor activity, RNA polymerase II-specific, actin filament binding, and chromatin binding. Additionally, the KEGG pathway analysis revealed that these genes were significantly involved in longevity regulating pathway, bile secretion, insulin secretion, and thyroid hormone signaling pathway (Fig. 3a and Additional file 3: Table S3). Furthermore, all terms were grouped into clusters based on the similarities, and a total of 13 clusters of significantly enriched terms were obtained (Fig. 3b), among these, sensory organ morphogenesis was the most enriched term.

### Establishment of PPI network

**Fig. 4:**
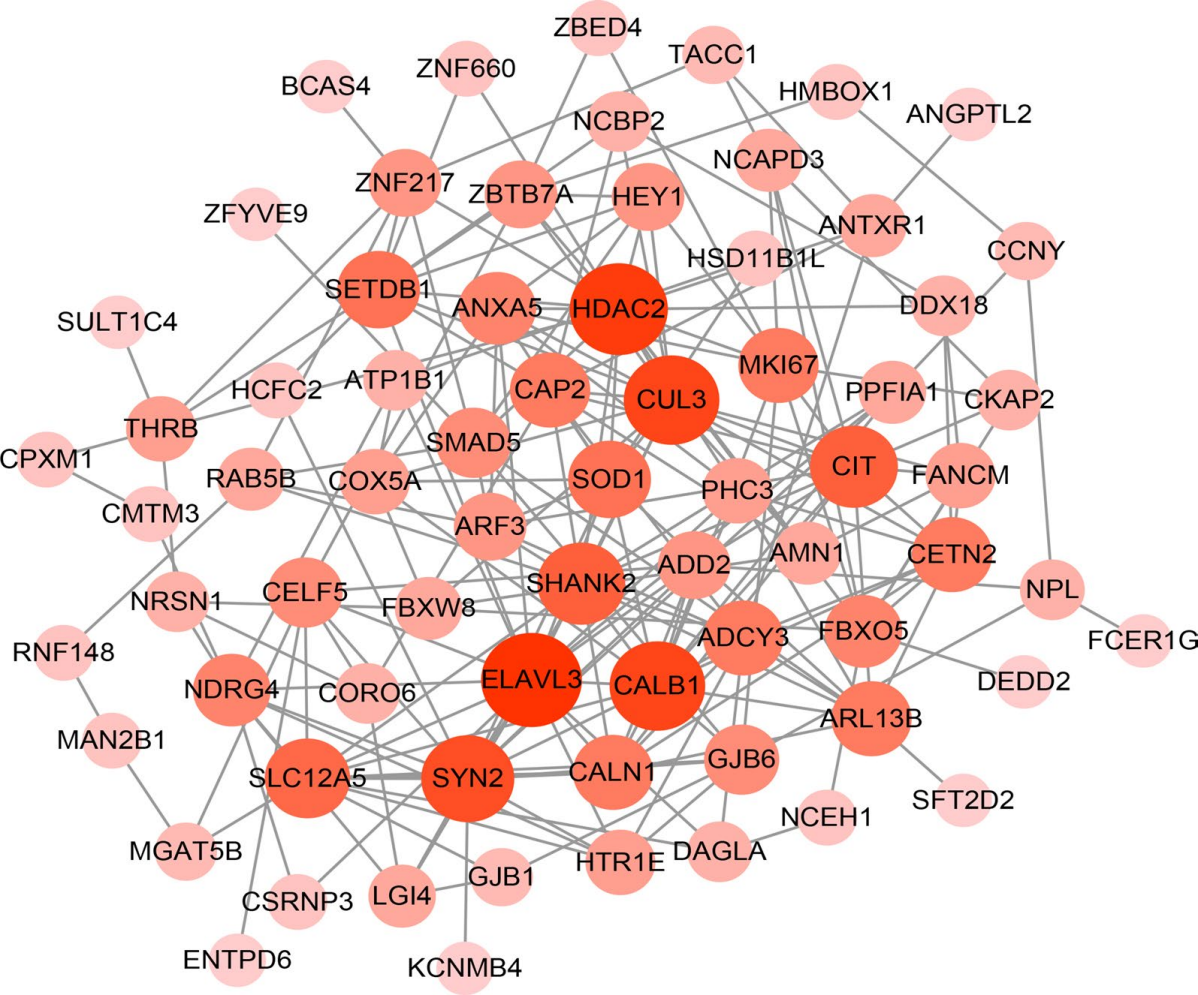
The PPI network of survival related DEGs. The color depth of nodes represents the corrected P-value. The size of nodes represents the number of genes involved

In order to understand the potential relationships between prognostic related DEGs, the PPI analysis was conducted. The PPI network was composed of 71 nodes and 214 edges (Fig. 4). A total of 16 nodes with the higher connectivity degrees were screened as hub genes, including ELAV like RNA binding protein 3 (*ELAVL3*), histone deacetylase 2 (*HDAC2*), Calbindin 1 (*CALB1*), cullin 3 (*CUL3*), synapsin II (*SYN2*), citron Rho-Interacting serine/threonine kinase (*CIT*), SH3 and multiple ankyrin repeat domains 2 (*SHANK2*), solute carrier family 12 member 5 (*SLC12A5*), superoxide dismutase 1 (*SOD1*), SET domain bifurcated histone lysine methyltransferase 1 (SETDB1), calneuron 1 (*CALN1*), cyclase associated actin cytoskeleton regulatory protein 2 (*CAP2*), ADP ribosylation factor like GTPase 13B (*ARL13B*), adenylate cyclase 3 (*ADCY3*), centrin 2 (*CETN2*), and marker of proliferation ki-67 (*MKI67*). Additionally, the specific degree values of these genes are listed in Table 2.

**Table 2:** The degree value of hub genes in PPI network

Symbol	Degree	Betweenness	Closeness
ELAVL3	18	649.4292	0.479452
HDAC2	17	758.398	0.486111
CALB1	16	283.0139	0.469799
CUL3	16	345.8947	0.479452
SYN2	15	363.5061	0.44586
CIT	13	246.3135	0.434783
SHANK2	13	332.2537	0.47619
SLC12A5	12	300.8864	0.4375
SOD1	11	222.0949	0.457516
SETDB1	11	338.4737	0.457516
CALN1	10	77.96878	0.434783
CAP2	10	185.884	0.434783
ARL13B	10	185.142	0.406977
ADCY3	10	160.4279	0.414201
CETN2	10	111.9884	0.414201
MKI67	10	247.3053	0.434783

### The mRNA level and mutation state of hub genes

**Fig. 5:**
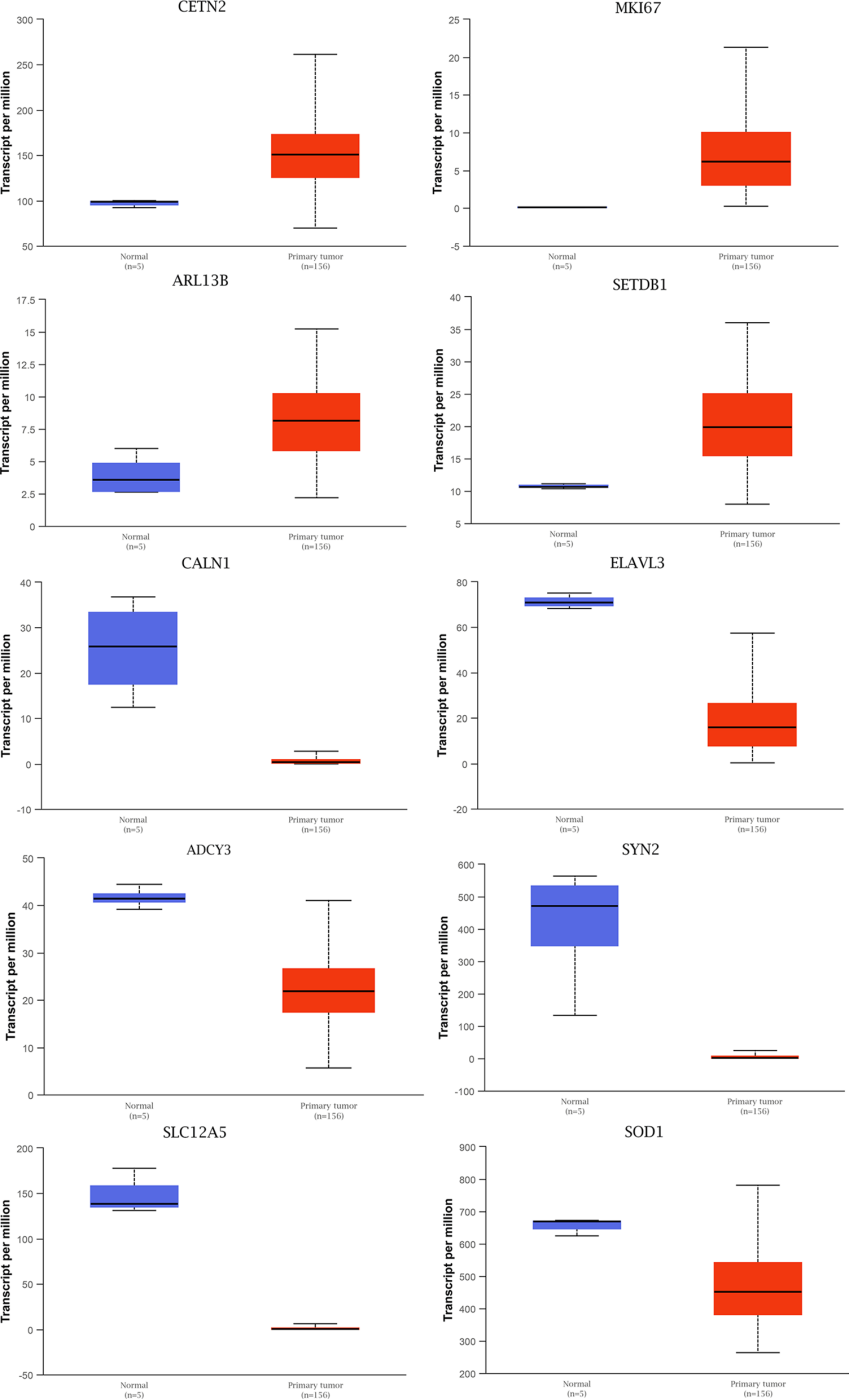
The mRNA expression level of hub genes according to the TCGA database. Blue box indicates normal tissue, and red box indicates GBM tissue

By analyzing the expression of the hub genes in the TCGA GBM data, we observed that the expression levels of 10 hub genes were consistent with the results of microarray datasets, including *MKI67*, *ARL13B*, *SETDB1*, *ELAVL3*, *ADCY3*, *SOD1*, *CALN1*, *SYN2*, and *SLC12A5*. Notably, compared with normal samples, the expression level of *MKI67*, *ARL13B*, and *SETDB1* was significantly up-regulated in GBM samples, while *ELAVL3*, *ADCY3*, *SOD1*, *CALN1*, *SYN2*, and *SLC12A5* were markedly down-regulated (Fig. 5 and Table 3). In addition, we also display the K-M curves of hub genes in Additional file 4: Fig. S1. Results showed that *CETN2*, *MKI67*, *ARL13B*, and *SETDB1* with lower expression level were related to a significantly longer survival time; meanwhile, high expression of *CALN1, ELAVL3, ADCY3, SYN2, ARL13B*, *SLC12A5*, and *SOD1* were associated with better overall survival of patients with GBM. The results of prognosis were consistent with the mRNA expression levels of hub genes.

**Table 3:** The expression level of hub genes

Genes	Comparison	Statistical significance	TCGA type	GEO type
MKI67	Normal-vs-Primary	1.62E−12	Up	Up
ELAVL3	Normal-vs-Primary	7.37E−12	Down	Down
ADCY3	Normal-vs-Primary	4.05E−06	Down	
CETN2	Normal-vs-Primary	8.01E−05	Up	
SOD1	Normal-vs-Primary	1.18E−03	Down	
ARL13B	Normal-vs-Primary	9.18E−03	Up	Up
CALN1	Normal-vs-Primary	2.39E−02	Down	Down
SYN2	Normal-vs-Primary	2.61E−02	Down	Down
SETDB1	Normal-vs-Primary	2.81E−02	Up	
SLC12A5	Normal-vs-Primary	4.31E−02	Down	Down

Furthermore, the hub gene mutations in GBM were tested using cBioPortal. The *MKI67*, *SLC12A5*, and *SOD1* exhibited higher mutation frequencies, and the proportion of them was 2.2, 0.7, and 0.2%, respectively (Additional file 5: Fig. S2A). Meanwhile, approximately 3% of GBM clinical cases showed significant alterations in the 10 hub genes (Additional file 5: Fig. S2B).

### Immunohistochemical analysis

**Fig. 6:**
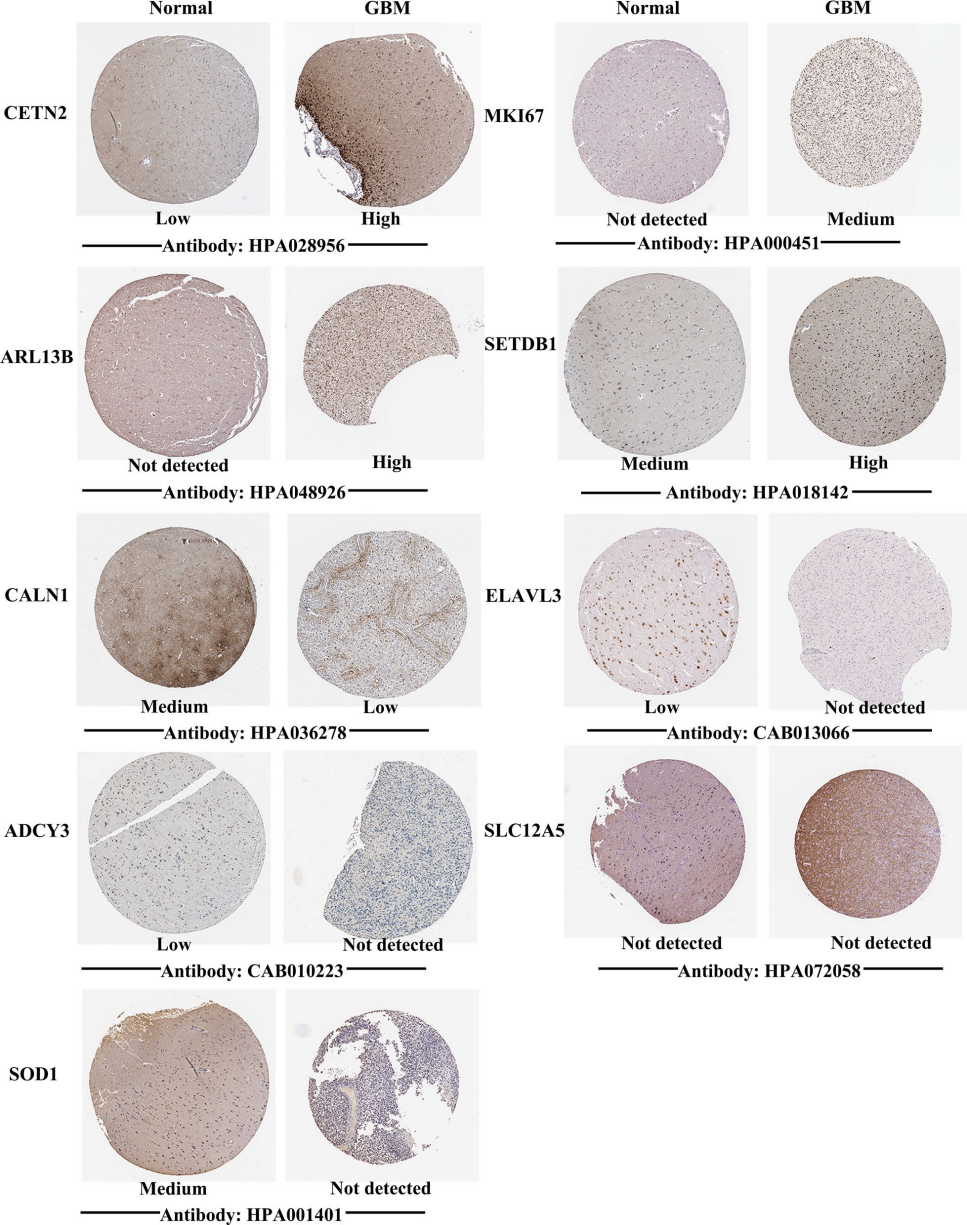
Immunohistochemistry images of hub genes in GBM tissues and normal tissues derived from the HPA database

Apart from investigating the mRNA level of hub genes, the protein expression levels were also explored using the HPA database. Because the immunohistochemical information of *SYN2* was not existed in HPA, we have displayed nine pairs of staining results in Fig. 6. The protein level of *MKI67* and *ARL13B* was undetected in normal tissues, while the level of these genes was medium and high in the GBM tissues, respectively. The protein level of *CETN2* was low in normal samples, while the level of it was high in GBM samples. Additionally, the medium protein level of *SETDB1* was observed in normal tissues, whereas the high protein level was revealed in GBM tissues. Meanwhile, the protein level of *CALN1* was medium in normal samples, while was low in the GBM samples. *SOD1* moderately expressed in normal tissues but undetectable in GBM tissues, and *ELAVL3* and *ADCY3* lowly expressed in normal tissues but undetectable in GBM tissues. Moreover, *SLC12A5* was undetectable in normal and GBM samples. Thus, *CETN2, MKI67*, *ARL13B*, *SETDB1*, and *CALN1* might be potential biomarkers for screening high-risk patients with GBM.

### Analysis of GBM-related small molecular drugs

**Fig. 7:**
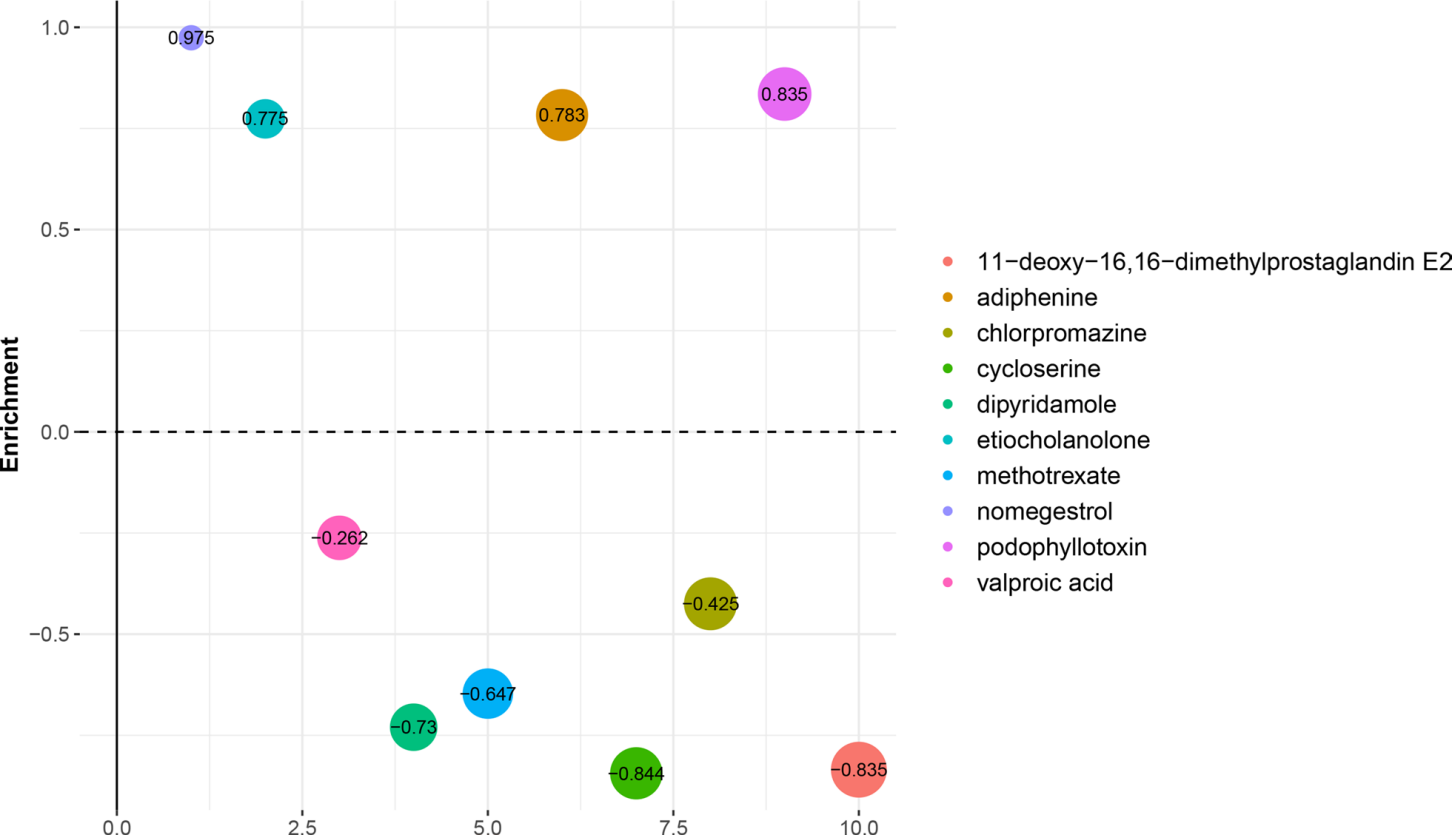
The top 10 small molecule drugs identified by CMAP database. The bubble size represents p value, the smaller the p value, the larger the bubble

To identify candidate small molecular drugs targeting the gene expression of GBM, all the prognosis-related DEGs were divided into up-regulated and down-regulated groups, which were submitted to the CMAP database. A total of 98 small molecular drugs that closely related to the biological status of GBM were obtained, of which 45 drugs might play potential synergies role in the development of GBM (enrichment score > 0), while 53 drugs might serve repress role in the GBM progression (enrichment score < 0). The top 10 vital small molecule drugs were selected (Fig. 7). Among these drugs, cycloserine (enrichment score = − 0.844) and 11-deoxy-16,16-dimethylprostaglandin E2 (enrichment score = − 0.835) showed highly significant negative correlation and had potential to reverse the carcinoma status of GBM. These identified small molecule drugs with enrichment scores < 0 could reverse the abnormal gene expression and serve as potential drugs for GBM treatment.

## Discussion

Although significant breakthrough in GBM treatment programs, including surgery, molecular therapy, and drug treatment, the prognosis for GBM patients remains poor and unchanged over the last 30 years [21]. Therefore, revealing the etiology and molecular mechanism of GBM might play important role in the diagnosis and treatment of tumor. In this study, bioinformatics analysis was used to screen the potential hub genes associated with GBM. By integration analysis of three GEO datasets of GBM, 613 overlapping DEGs were identified, among these, 78 DEGs were significantly associated with the OS of GBM. The GO analysis showed that these DEGs was mainly enriched in trans-synaptic signaling; and the KEGG pathways enrichment analysis indicated that DEGs were significantly involved in longevity regulating pathway. PPI analysis revealed that *CETN2*, *MKI67*, *ARL13B*, *SETDB1*, *CALN1*, *ELAVL3*, *ADCY3*, *SYN2*, *SLC12A5*, and *SOD1* with high degree of connectivity were selected as hub genes. For *CETN2*, *MKI67*, *ARL13B*, and *SETDB1*, patients with high expression experienced a worse OS, while high expression of *CALN1, ELAVL3, ADCY3, SYN2, ARL13B*, *SLC12A5*, and *SOD1* were associated with better overall survival among patients with GBM. To validate the results of bioinformatics analysis, we evaluated the mRNA and protein expression levels of hub genes by using TCGA and HPA databases. The results showed the same gene expression trend as observed in the GEO database, which further confirmed the accuracy of our findings. Specially, *CETN2, MKI67*, *ARL13B*, *SETDB1*, and *CALN1* might be potential biomarkers for screening high-risk patients with GBM. Furthermore, the small molecular drugs analysis showed that cycloserine and 11-deoxy-16,16-dimethylprostaglandin E2 might as potential therapeutic drugs for GBM.

In this study, GO analysis revealed that trans-synaptic signaling was the significantly enriched term for DEGs, which was consistent with previous study [22]. During the process of synaptogenesis, the glycans could modulate trans-synaptic signaling [23]; interestingly, glycans served important role in cancer progression and treatment. Glycosylation resulted in a variety of functional changes in glycoproteins, including adhesion molecules and cell surface receptors, such as e-cadherin and integrin. Notably, these changes conferred distinctive phenotypic characteristics connected with cancer cells [24]. Bassoy et al. observed that the sensitivity of glioma cells to cytotoxic lymphocytes might increase with the decrease of glycan surface expression [25]. Besides, metabotropic glutamate receptors, which involved in synaptic signaling, also participated in the transformation of multiple cancer types, such as GBM, breast cancer, and melanoma skin cancer [26]. These findings suggested that trans-synaptic signaling might play a vital role in the pathogenesis of GBM.

PPI analysis showed that *CETN2* was a hub gene, as well as the mRNA and protein expression levels of it were over-expressed in the GBM tissues. *CETN2* was a member of the calcium-binding protein family, and caltractin played a fundamental role in structure and function of the microtubule-organizing center [27]. In addition, *CETN2* was also involved in nucleotide excision repair that was linked with the risk of cancer [28]. Accumulating evidences demonstrated that *CETN2* was identified in various types of cancers. Huan et al. revealed that *CETN2* was associated with invasive ductal carcinoma of the breast, and might be potential biomarker for breast cancer [29]. It was reported that the down-regulated of *CETN2* might have tumor suppressive function in bladder cancer [30]. Similarly, we found that the low expression level of *CETN2* was significantly related to better survival of GBM patients. However, no studies have reported the potential mechanism of *CETN2* in the initiation and progression of GBM. Hence, the mechanism of how *CETN2* contributed to the GBM still need further research.

Meanwhile, we also found *MKI67* was closely related to the prognosis of GBM. The protein encoded by *MKI67* was necessary for cellular proliferation. Hou et al. showed that the down-regulated of *MKI67* could suppress cell growth in the hepatocellular carcinoma cell [31]. Laible et al. indicated that *MKI67* was a biomarker of breast cancer [32]. Meanwhile, *MKI67* was connected with nuclear features and the survival of GBM [33, 34]. In this study, *MKI67* was up-regulated in GBM tissues, and GBM patients with a low *MKI67* expression level displayed longer survival. Györffy et al. showed *MKI67* was a prognostic factor in breast carcinoma [35]. Taken together, we speculated that *MKI67* played vital roles in GBM progression and might serve as a molecular target for GBM treatment.

*ARL13B* was also involved in the GBM development, and the protein level of *ARL13B* was higher in tumor samples than in normal samples. Casalou et al. confirmed that breast cancer was promoted by *ARL13B*, which was connected with cancer cell migration and invasion [36]. Another gene, *SETDB1* regulated histone methylation, gene silencing, and transcriptional repression [37]. *SETDB1* mediated Akt methylation promoted its k63-linked ubiquitination and activation, leading to tumorigenesis [38]. Meanwhile, the oncogenic role of *SETDB1* has been reported in GBM [39], which was further supported our findings. In addition, *CALN1* was another hub gene in PPI analysis. *CALN1* encoded a protein with high similarity to the calcium-binding proteins of the calmodulin family. *CALN1* might influence the invasion and migration of osteosarcoma cell line, and it was also associated with the survival of osteosarcoma [40]. We found the high expression level of *CALN1* was related to the poor prognosis of GBM. *ELAVL3* was one of neuronal-specific RNA-binding proteins (Hu antigens), which was recognized by anti-hu antibody in the serum of patients with paraneoplastic encephalomyelitis and sensory neuropathy [41]. Delgado-López et al. revealed that the expression of *ELAVL3* was increased in the GBM tissues [42]. Unfortunately, we found *ELAVL3* was down-regulated in the tumor samples, this discrepancy required further study. *ADCY3* could catalyze the formation of cyclic adenosine monophosphate [43]. Hong et al. indicated that *ADCY3* was overexpressed in the gastric cancer tissues and promoted cell proliferation, migration, as well as invasion [44]. In this study, we found the high expression level of *ADCY3* with worse overall survival. Additionally, we observed *SLC12A5* was closely involved in the development of GBM. Verhaak et al. found that *SLC12A5* was a common biomarker of GBM [45], which was consistent with our results. Furthermore, we observed that *SOD1* was closely relevant to the prognosis of GBM. The protein encoded by *SOD1* bound copper and zinc ions, and *SOD1* was responsible for destroying superoxide free radicals in the body. Kato et al. demonstrated that the expression level of *SOD1* was significantly changed in the GBM [46]. Gao et al. indicated that GBM with low expression level of *SOD1* had better response to radiotherapy [47]. In the present study, patients with low expression of *SOD1* experienced a better prognosis. Taken together, these genes served vital role in the development of GBM. However, our study was performed based on the bioinformatics analysis, further experimental studies must be conducted to understand the potential effect of key genes in the GBM pathogenesis.

Based on the small molecular drugs analysis, we determined a set of small molecule drugs that had potential to reverse the abnormal gene expression changes of GBM. Among these, cycloserine and 11-deoxy-16,16-dimethylprostaglandin E2 showed highly significant negative correlation and might serve as potential drugs for GBM treatment. Cycloserine is a cyclic analog to d-alanine, which can target alanine racemase and d-alanine ligase, thereby preventing the formation of bacterial cell walls [48]. Recently, cycloserine has been widely used in tuberculosis treatment, but no research has focused on the potential role of it in GBM. In addition, previous study revealed that 11-deoxy-16,16-dimethylprostaglandin E2 (DDM-PGE2) protected proximal renal tubular epithelial cells from potent nephrotoxicity-induced cell damage by exerting anti-oxidative stress [49]. Meanwhile, it also protected against oncotic cell death which induced by H(2)O(2) and iodoacetamide [50]. Similarly, the relationship between DDM-PGE2 and GBM was not investigated. Given the emergence of these small molecules drugs in silico, further studies that explore the potential effects of them on GBM are imperative and will contribute to the study on new therapeutic drugs for GBM.

Despite studies devoted to investigate the molecular mechanisms of GBM development, integrated studies based on multiple datasets are rare. In the present study, 10 hub genes were identified for the first time in GBM by integrated bioinformatics analysis; meanwhile, the mRNA and protein expression levels of them were verified by using TCGA and HPA databases. Importantly, we also screened the putative therapeutic agents for GBM. This study comprehensively analyzed the pathogenesis of GBM, which provided certain guiding significance for the diagnosis and treatment of this disease. Although the clinical value of these genes and drugs in GBM has not been reported in previous study, the importance of them should not be underestimated.

## Conclusions

In summary, with the integrated bioinformatics analysis of three GBM-related gene expression profiles, we identified 10 key genes connected with pathogenesis and prognosis of GBM. These hub genes might serve as novel diagnostic and treatment biomarkers of GBM, which might conduct to elucidate the molecular mechanism of the occurrence and progression of GBM. Additionally, a series of small molecule drugs which could reverse the abnormal gene expression of GBM were identified. Our work may provide powerful evidence for the genomic individualized treatment of GBM.

## Supplementary information

**Additional file 1: Table S1.** The number of DEGs obtained from three datasets.

**Additional file 2: Table S2.** The prognosis-relate differentially expressed genes.

**Additional file 3: Table S3.** Functional and pathway enrichment analysis of prognostic related DEGs.

**Additional file 4: Fig.** **S1**. Survival analysis for hub genes in GBM. Kaplan–Meier plots show 10 hub genes related to overall survival rate (P < 0.05). A: CETN2, B: MKI67, C: ARL13B, D: SETDB1, E: CALN1, F: ELAVL3, G: ADCY3, H: SYN2, I: SLC12A5, J: SOD1.

**Additional file 5: Fig.** **S2.** Gene mutation frequencies of hub genes. A: The mRNA alterations of hub genes. The dark blue bars represent deep deletion, the pink bars represent mRNA up-regulation, the pool blue bars represent mRNA down-regulation, and gray bars represent no alteration. B: Percentage of gene mutations in GBM patients.

## Data Availability

The data that support the findings of this study are available from University of California Santa Cruz Genome Browser and GEO database.

## Abbreviations

GBM: Glioblastoma
ESR2: Estrogen receptor 2
ELOVL6: ELOVL fatty acid elongase 6
IRX3: Iroquois homeobox 3
AhR: Aryl hydrocarbon receptor
GSCs: GBM stem cells
PBK: PDZ binding kinase
CENPA: Centromere protein A
KIF15: Kinesin family member 15
GEO: Gene Expression Omnibus
TCGA: The Cancer Genome Atlas
PPI: Protein-protein interaction
CMAP: Connectivity map
RMA: Robust multi-array average
FC: Fold change
GDC: Genomic data commons
OS: Overall survival
KM: Kaplan–Meier
GO: Gene ontology
HPAs: Human protein atlas
CETN2: Centrin 2
MKI67: Marker of proliferation ki-67
ARL13B: ADP ribosylation factor like GTPase 13B
SETDB1: SET domain bifurcated histone lysine methyltransferase 1
CALN1: Calneuron 1
ELAVL3: ELAV like RNA binding protein 3
ADCY3: Adenylate cyclase 3
SYN2: Synapsin II
SLC12A5: Solute carrier family 12 member 5
SOD1: Superoxide dismutase 1

## References

[1] Lukas RV, Rodon J, Becker K, Wong ET, Shih K, Touat M (2018). Clinical activity and safety of atezolizumab in patients with recurrent glioblastoma. J Neurooncol.

[2] Alexander BM, Cloughesy TF (2017). Adult glioblastoma. J Clin Oncol.

[3] Delgado-López P, Corrales-García E (2016). Survival in glioblastoma: a review on the impact of treatment modalities. Clin Transl Oncol.

[4] Shergalis A, Bankhead A, Luesakul U, Muangsin N, Neamati N (2018). Current challenges and opportunities in treating glioblastoma. Pharmacol Rev.

[5] Stangeland B, Mughal AA, Grieg Z, Sandberg CJ, Joel M, Nygård S (2015). Combined expressional analysis, bioinformatics and targeted proteomics identify new potential therapeutic targets in glioblastoma stem cells. Oncotarget..

[6] Lu G, Rao M, Zhu P, Liang B, El-Nazer RT, Fonkem E (2019). Triple-drug therapy with bevacizumab, irinotecan, and temozolomide plus tumor treating fields for recurrent glioblastoma: a retrospective study. Front Neurol..

[7] Barrett T, Suzek TO, Troup DB, Wilhite SE, Ngau W-C, Ledoux P (2005). NCBI GEO: mining millions of expression profiles—database and tools. Nucleic Acids Res..

[8] Barrett T, Troup DB, Wilhite SE, Ledoux P, Rudnev D, Evangelista C (2006). NCBI GEO: mining tens of millions of expression profiles—database and tools update. Nucleic Acids Res..

[9] Ritchie ME, Phipson B, Wu D, Hu Y, Law CW, Shi W (2015). Limma powers differential expression analyses for RNA-sequencing and microarray studies. Nucleic Acids Res.

[10] Bolstad BM, Irizarry RA, Åstrand M, Speed TP (2003). A comparison of normalization methods for high density oligonucleotide array data based on variance and bias. Bioinformatics.

[11] Irizarry RA, Hobbs B, Collin F, Beazer-Barclay YD, Antonellis KJ, Scherf U (2003). Exploration, normalization, and summaries of high density oligonucleotide array probe level data. Biostatistics..

[12] Zhou G, Soufan O, Ewald J, Hancock RE, Basu N, Xia J (2019). NetworkAnalyst 3.0: a visual analytics platform for comprehensive gene expression profiling and meta-analysis. Nucleic Acids Res..

[13] Goldman M, Craft B, Hastie M, Repecka K, Kamath A, McDade F (2019). The UCSC Xena platform for public and private cancer genomics data visualization and interpretation. bioRxiv..

[14] Zhou Y, Zhou B, Pache L, Chang M, Khodabakhshi AH, Tanaseichuk O (2019). Metascape provides a biologist-oriented resource for the analysis of systems-level datasets. Nat Commun..

[15] Szklarczyk D, Morris JH, Cook H, Kuhn M, Wyder S, Simonovic M (2016). The STRING database in 2017: quality-controlled protein–protein association networks, made broadly accessible. Nucleic Acids Res.

[16] Shannon P, Markiel A, Ozier O, Baliga NS, Wang JT, Ramage D (2003). Cytoscape: a software environment for integrated models of biomolecular interaction networks. Genome Res.

[17] Chandrashekar DS, Bashel B, Balasubramanya SAH, Creighton CJ, Ponce-Rodriguez I, Chakravarthi BV (2017). UALCAN: a portal for facilitating tumor subgroup gene expression and survival analyses. Neoplasia..

[18] Gao J, Aksoy BA, Dogrusoz U, Dresdner G, Gross B, Sumer SO (2013). Integrative analysis of complex cancer genomics and clinical profiles using the cBioPortal. Sci Signal..

[19] Uhlen M, Zhang C, Lee S, Sjöstedt E, Fagerberg L, Bidkhori G (2017). A pathology atlas of the human cancer transcriptome. Science..

[20] Subramanian A, Narayan R, Corsello SM, Peck DD, Natoli TE, Lu X (2017). A next generation connectivity map: L1000 platform and the first 1,000,000 profiles. Cell..

[21] Hanif F, Muzaffar K, Perveen K, Malhi SM, Simjee SU (2017). Glioblastoma multiforme: a review of its epidemiology and pathogenesis through clinical presentation and treatment. APJCP..

[22] Yang S, Gao K, Li W (2019). Identification of hub genes and pathways in glioblastoma by bioinformatics analysis. Oncol Lett..

[23] Dani N, Broadie K (2012). Glycosylated synaptomatrix regulation of trans-synaptic signaling. Dev Neurobiol..

[24] Taniguchi N, Kizuka Y (2015). Glycans and cancer: role of N-glycans in cancer biomarker, progression and metastasis, and therapeutics. Adv Cancer Res.

[25] Bassoy EY, Kasahara A, Chiusolo V, Jacquemin G, Boydell E, Zamorano S (2017). ER–mitochondria contacts control surface glycan expression and sensitivity to killer lymphocytes in glioma stem-like cells. EMBO J..

[26] Lumeng JY, Wall BA, Wangari-Talbot J, Chen S (2017). Metabotropic glutamate receptors in cancer. Neuropharmacology.

[27] Krasikova YS, Rechkunova N, Maltseva E, Craescu C, Petruseva I, Lavrik O (2012). Influence of centrin 2 on the interaction of nucleotide excision repair factors with damaged DNA. Biochemistry.

[28] Kamileri I, Karakasilioti I, Garinis GA (2012). Nucleotide excision repair: new tricks with old bricks. Trends Genet.

[29] Huan J, Gao X, Xing L, Qin X, Qian H, Zhou Q (2014). Screening for key genes associated with invasive ductal carcinoma of the breast via microarray data analysis. Genet Mol Res.

[30] Tatarano S, Chiyomaru T, Kawakami K, Enokida H, Yoshino H, Hidaka H (2011). miR-218 on the genomic loss region of chromosome 4p15. 31 functions as a tumor suppressor in bladder cancer. Int J Oncol..

[31] Hou Y-Y, Cao W-W, Li L, Li S-P, Liu T, Wan H-Y (2011). MicroRNA-519d targets MKi67 and suppresses cell growth in the hepatocellular carcinoma cell line QGY-7703. Cancer Lett.

[32] Laible M, Schlombs K, Kaiser K, Veltrup E, Herlein S, Lakis S (2016). Technical validation of an RT-qPCR in vitro diagnostic test system for the determination of breast cancer molecular subtypes by quantification of ERBB2, ESR1, PGR and MKI67 mRNA levels from formalin-fixed paraffin-embedded breast tumor specimens. BMC Cancer..

[33] Wang R-j, Li J-w, Bao B-h, Wu H-c, Du Z-h, Su J-l (2015). MicroRNA-873 (miRNA-873) inhibits glioblastoma tumorigenesis and metastasis by suppressing the expression of IGF2BP1. J Biol Chem.

[34] Kong J, Wang F, Teodoro G, Cooper L, Moreno CS, Kurc T (2013). High-performance computational analysis of glioblastoma pathology images with database support identifies molecular and survival correlates. Proceedings IEEE Int Conf Bioinformatics Biomed..

[35] Györffy B, Lanczky A, Eklund AC, Denkert C, Budczies J, Li Q (2010). An online survival analysis tool to rapidly assess the effect of 22,277 genes on breast cancer prognosis using microarray data of 1,809 patients. Breast Cancer Res Treat.

[36] Casalou C, Faustino A, Silva F, Ferreira IC, Vaqueirinho D, Ferreira A (2019). Arl13b regulates breast cancer cell migration and invasion by controlling integrin-mediated signaling. Cancers..

[37] Fuks F (2005). DNA methylation and histone modifications: teaming up to silence genes. Curr Opin Genet Dev.

[38] Wang G, Long J, Gao Y, Zhang W, Han F, Xu C (2019). SETDB1-mediated methylation of Akt promotes its K63-linked ubiquitination and activation leading to tumorigenesis. Nat Cell Biol.

[39] Spyropoulou A, Gargalionis A, Dalagiorgou G, Adamopoulos C, Papavassiliou KA, Lea RW (2014). Role of histone lysine methyltransferases SUV39H1 and SETDB1 in gliomagenesis: modulation of cell proliferation, migration, and colony formation. NeuroMol Med.

[40] Gong L, Bao Q, Hu C, Wang J, Zhou Q, Wei L (2018). Exosomal miR-675 from metastatic osteosarcoma promotes cell migration and invasion by targeting CALN1. Biochem Biophys Res Commun.

[41] Pignolet BS, Gebauer CM, Liblau RS (2013). Immunopathogenesis of paraneoplastic neurological syndromes associated with anti-Hu antibodies: a beneficial antitumor immune response going awry. Oncoimmunology..

[42] Delgado-López PD, Corrales-García EM (2016). Survival in glioblastoma: a review on the impact of treatment modalities. Clin Translatl Oncol..

[43] Goni L, Riezu-Boj JI, Milagro FI, Corrales FJ, Ortiz L, Cuervo M (2018). Interaction between an ADCY3 genetic variant and two weight-lowering diets affecting body fatness and body composition outcomes depending on macronutrient distribution: a randomized trial. Nutrients..

[44] Hong S-H, Goh S-H, Lee SJ, Hwang J-A, Lee J, Choi I-J (2013). Upregulation of adenylate cyclase 3 (ADCY3) increases the tumorigenic potential of cells by activating the CREB pathway. Oncotarget..

[45] Labak CM, Wang PY, Arora R, Guda MR, Asuthkar S, Tsung AJ (2016). Glucose transport: meeting the metabolic demands of cancer, and applications in glioblastoma treatment. Am J Cancer Res..

[46] Kato S, Esumi H, Hirano A, Kato M, Asayama K, Ohama E (2003). Immunohistochemical expression of inducible nitric oxide synthase (iNOS) in human brain tumors: relationships of iNOS to superoxide dismutase (SOD) proteins (SOD1 and SOD2), Ki-67 antigen (MIB-1) and p53 protein. Acta Neuropathol.

[47] Gao Z, Sarsour EH, Kalen AL, Li L, Kumar MG, Goswami PC (2008). Late ROS accumulation and radiosensitivity in SOD1-overexpressing human glioma cells. Free Radical Biol Med.

[48] Li Y, Wang F, Wu L, Zhu M, He G, Chen X (2019). Cycloserine for treatment of multidrug-resistant tuberculosis: a retrospective cohort study in China. Infect Drug Resist.

[49] Towndrow KM, Jia Z, Lo HH, Person MD, Monks TJ, Lau SS (2003). 11-Deoxy,16,16-dimethyl prostaglandin E2 induces specific proteins in association with its ability to protect against oxidative stress. Chem Res Toxicol.

[50] Jia Z, Person MD, Dong J, Shen J, Hensley SC, Stevens JL (2004). Grp78 is essential for 11-deoxy-16,16-dimethyl PGE2-mediated cytoprotection in renal epithelial cells. Am J Physiol Renal Physiol.

