# Supplementary material for: Identification of potential biomarkers and candidate small molecule drugs in glioblastoma
Source: Cancer Cell Int. 2020 Aug 28;20:419. doi: 10.1186/s12935-020-01515-1 (PMC7455906; doi:10.1186/s12935-020-01515-1)
Supplement: Supplementary file 1 — Additional file 1: Table S1. The number of DEGs obtained from three datasets. [file 12935_2020_1515_MOESM1_ESM.docx]

Additional file 1: Table S1 The number of DEGs obtained from three datasets

|  | GSE103227 | GSE104267 | GSE111260 |
| --- | --- | --- | --- |
| Up-regulated genes | 1834 | 586 | 632 |
| Down-regulated genes | 1832 | 454 | 877 |
| Total | 3666 | 1040 | 1509 |
