# Supplementary material for: Identification of potential biomarkers and candidate small molecule drugs in glioblastoma
Source: Cancer Cell Int. 2020 Aug 28;20:419. doi: 10.1186/s12935-020-01515-1 (PMC7455906; doi:10.1186/s12935-020-01515-1)
Supplement: Supplementary file 2 — Additional file 2: Table S2. The prognosis-relate differentially expressed genes. [file 12935_2020_1515_MOESM2_ESM.docx]

Additional file 2: Table S2 The prognosis-relate differentially expressed genes

| Gene | P value | High.median | Low.median | Gene | P value | High.median | Low.median |
| --- | --- | --- | --- | --- | --- | --- | --- |
| CKAP2 | 0.00004 | 12.00 | 16.87 | FANCM | 0.02354 | 12.80 | 14.73 |
| ZNF660 | 0.00019 | 12.00 | 16.17 | SLC12A5 | 0.02356 | 12.73 | 15.17 |
| SOD1 | 0.00031 | 12.83 | 15.33 | CMTM3 | 0.02357 | 16.17 | 12.07 |
| TMEM69 | 0.00090 | 12.00 | 15.33 | PHC3 | 0.02366 | 12.93 | 15.33 |
| MANSC1 | 0.00094 | 15.60 | 12.07 | ABR | 0.02512 | 15.33 | 12.73 |
| NCAPD3 | 0.00156 | 15.33 | 12.50 | NCBP2 | 0.02643 | 14.93 | 12.80 |
| CETN2 | 0.00204 | 12.93 | 16.17 | ADCY3 | 0.02687 | 12.93 | 15.13 |
| ATP1B1 | 0.00285 | 12.73 | 15.13 | ANTXR1 | 0.02737 | 12.93 | 15.13 |
| NDRG4 | 0.00313 | 12.00 | 16.83 | SMAD5 | 0.02763 | 13.13 | 15.13 |
| CORO6 | 0.00343 | 16.17 | 12.50 | HCFC2 | 0.02821 | 12.50 | 15.33 |
| TMEM121 | 0.00370 | 16.17 | 12.80 | SHANK2 | 0.02898 | 12.80 | 14.93 |
| CSRNP3 | 0.00396 | 12.80 | 16.00 | PPFIA1 | 0.02946 | 12.67 | 16.17 |
| ZFYVE9 | 0.00400 | 12.50 | 16.00 | SCAF11 | 0.03057 | 16.17 | 12.80 |
| TACC1 | 0.00412 | 12.00 | 15.60 | THRB | 0.03150 | 12.67 | 15.33 |
| FBXO5 | 0.00416 | 16.87 | 12.07 | DAGLA | 0.03153 | 16.17 | 12.93 |
| CPXM1 | 0.00509 | 16.00 | 12.07 | CCNY | 0.03226 | 14.93 | 13.30 |
| DDX18 | 0.00690 | 11.40 | 16.00 | ELAVL3 | 0.03361 | 14.23 | 13.50 |
| NCEH1 | 0.00701 | 12.07 | 16.00 | ANXA5 | 0.03397 | 14.23 | 12.93 |
| SULT1C4 | 0.00805 | 12.07 | 15.33 | HSD11B1L | 0.03490 | 13.97 | 13.53 |
| CAP2 | 0.00966 | 12.67 | 16.17 | SYN2 | 0.03529 | 13.30 | 15.13 |
| DEDD2 | 0.00997 | 15.13 | 13.13 | KCNMB4 | 0.03550 | 12.07 | 15.13 |
| HEY1 | 0.01038 | 12.07 | 15.33 | ZBED4 | 0.03631 | 15.17 | 12.93 |
| ANGPTL2 | 0.01091 | 12.50 | 15.33 | MGAT5B | 0.03645 | 13.13 | 14.40 |
| MKI67 | 0.01094 | 12.83 | 15.33 | COX5A | 0.03684 | 14.23 | 13.80 |
| HMBOX1 | 0.01234 | 15.33 | 12.50 | SETDB1 | 0.03741 | 14.93 | 12.83 |
| CELF5 | 0.01250 | 12.83 | 16.17 | CDV3 | 0.03753 | 14.93 | 12.83 |
| GJB6 | 0.01347 | 12.50 | 15.60 | NRSN1 | 0.03759 | 15.13 | 12.73 |
| CIT | 0.01571 | 15.33 | 12.50 | ARF3 | 0.03813 | 12.00 | 15.60 |
| RNF148 | 0.01757 | 12.80 | 15.17 | ZBTB7A | 0.03822 | 12.93 | 14.40 |
| CALB1 | 0.01795 | 14.93 | 12.80 | RAB5B | 0.03833 | 12.80 | 15.33 |
| ENTPD6 | 0.01857 | 12.50 | 15.17 | ARL13B | 0.03988 | 15.17 | 12.80 |
| GJB1 | 0.01874 | 12.73 | 15.60 | FBXW8 | 0.04399 | 12.50 | 16.00 |
| CUL3 | 0.01878 | 12.67 | 16.17 | TSPAN5 | 0.04425 | 14.93 | 12.93 |
| FCER1G | 0.01901 | 15.33 | 12.93 | CALN1 | 0.04439 | 13.13 | 15.13 |
| BCAS4 | 0.01924 | 12.93 | 16.00 | HDAC2 | 0.04699 | 12.07 | 15.33 |
| HTR1E | 0.01968 | 12.00 | 15.13 | AMN1 | 0.04760 | 14.73 | 12.83 |
| MAN2B1 | 0.02118 | 15.60 | 12.07 | ADD2 | 0.04789 | 15.33 | 12.73 |
| SFT2D2 | 0.02226 | 15.33 | 13.13 | NPL | 0.04822 | 12.83 | 15.13 |
| ZNF217 | 0.02328 | 13.30 | 15.13 | LGI4 | 0.04873 | 14.93 | 12.80 |
