# Supplementary material for: Identification of potential biomarkers and candidate small molecule drugs in glioblastoma
Source: Cancer Cell Int. 2020 Aug 28;20:419. doi: 10.1186/s12935-020-01515-1 (PMC7455906; doi:10.1186/s12935-020-01515-1)
Supplement: Supplementary file 3 — Additional file 3: Table S3. Functional and pathway enrichment analysis of prognostic related DEGs. [file 12935_2020_1515_MOESM3_ESM.doc]

Additional file Table S3 Functional and pathway enrichment analysis of prognostic related DEGs

| Category | Terms | Count | Genes | LogP |
| --- | --- | --- | --- | --- |
| GO_BP | GO:0007610 behavior | 10 | ADCY3|CALB1|HDAC2|MAN2B1|SOD1|THRB|SHANK2|SLC12A5|NDRG4|LGI4 | -4.5 |
| GO_BP | GO:0007611 learning or memory | 6 | ADCY3|CALB1|MAN2B1|SHANK2|SLC12A5|NDRG4 | -3.7 |
| GO_BP | GO:0090596 sensory organ | 6 | ABR|CALB1|HDAC2|SOD1|THRB|GJB6 | -3.7 |
| GO_BP | GO:0051304 chromosome separation | 4 | CUL3|NCAPD3|FBXO5|FANCM | -3.6 |
| GO_BP | GO:0050890 cognition | 6 | ADCY3|CALB1|MAN2B1|SHANK2|SLC12A5|NDRG4 | -3.4 |
| GO_BP | GO:0099537 trans-synaptic signaling | 9 | ABR|DAGLA|CALB1|HTR1E|SYN2|PPFIA1|SHANK2|KCNMB4|SLC12A5 | -3.3 |
| GO_BP | GO:0099536 synaptic signaling | 9 | ABR|DAGLA|CALB1|HTR1E|SYN2|PPFIA1|SHANK2|KCNMB4|SLC12A5 | -3.2 |
| GO_BP | GO:0048568 embryonic organ development | 7 | ABR|SOD1|GJB6|HEY1|FBXW8|NDRG4|ARL13B | -3.2 |
| GO_BP | GO:0051301 cell division | 8 | CETN2|TACC1|CUL3|CIT|NCAPD3|FBXO5|CKAP2|CCNY | -3.1 |
| GO_BP | GO:0007612 learning | 4 | ADCY3|SHANK2|SLC12A5|NDRG4 | -2.9 |
| GO_BP | GO:0050766 positive regulation of phagocytosis | 3 | ABR|FCER1G|SOD1 | -2.9 |
| GO_BP | GO:0002262 myeloid cell homeostasis | 4 | FCER1G|SMAD5|SOD1|ZBTB7A | -2.9 |
| GO_BP | GO:0030036 actin cytoskeleton organization | 8 | ABR|ADD2|CUL3|PPFIA1|CAP2|CIT|ANTXR1|CORO6 | -2.8 |
| GO_BP | GO:0071407 cellular response to organic cyclic compound | 7 | ADCY3|HDAC2|SMAD5|SOD1|THRB|DDX18|ZBTB7A | -2.7 |
| GO_BP | GO:0007268 chemical synaptic transmission | 8 | ABR|CALB1|HTR1E|SYN2|PPFIA1|SHANK2|KCNMB4|SLC12A5 | -2.6 |
| GO_BP | GO:0098916 anterograde trans-synaptic signaling | 8 | ABR|CALB1|HTR1E|SYN2|PPFIA1|SHANK2|KCNMB4|SLC12A5 | -2.6 |
| GO_BP | GO:0000910 cytokinesis | 4 | CETN2|CUL3|CIT|CKAP2 | -2.6 |
| GO_BP | GO:0048562 embryonic organ morphogenesis | 5 | ABR|SOD1|GJB6|NDRG4|ARL13B | -2.5 |
| GO_BP | GO:0032465 regulation of cytokinesis | 3 | CETN2|CUL3|CIT | -2.5 |
| GO_BP | GO:0048598 embryonic morphogenesis | 7 | ABR|HDAC2|SOD1|CUL3|GJB6|NDRG4|ARL13B | -2.5 |
| GO_BP | GO:0050764 regulation of phagocytosis | 3 | ABR|FCER1G|SOD1 | -2.4 |
| GO_BP | GO:0030029 actin filament-based process | 8 | ABR|ADD2|CUL3|PPFIA1|CAP2|CIT|ANTXR1|CORO6 | -2.4 |
| GO_BP | GO:0048839 inner ear development | 4 | ABR|CALB1|SOD1|GJB6 | -2.4 |
| GO_BP | GO:1990778 protein localization to cell periphery | 5 | ATP1B1|FCER1G|PPFIA1|TSPAN5|ARL13B | -2.4 |
| GO_BP | GO:0060996 dendritic spine development | 3 | HDAC2|SHANK2|SLC12 | -2.4 |
| GO_BP | GO:0007059 chromosome segregation | 5 | MKI67|CUL3|NCAPD3|FBXO5|FANCM | -2.3 |
| GO_BP | GO:0051983 regulation of chromosome segregation | 3 | MKI67|CUL3|FBXO5 | -2.3 |
| GO_BP | GO:0008593 regulation of Notch signaling pathway | 3 | TSPAN5|HEY1|ZBTB7A | -2.3 |
| GO_BP | GO:0043583 ear development | 4 | ABR|CALB1|SOD1|GJB6 | -2.2 |
| GO_BP | GO:0033044 regulation of chromosome organization | 5 | MKI67|CUL3|SETDB1|FBXO5|HMBOX1 | -2.2 |
| GO_BP | GO:0051098 regulation of binding | 5 | ADD2|HDAC2|HEY1|ZBTB7A|HMBOX1 | -2.2 |
| GO_BP | GO:0097435 supramolecular fiber organization | 7 | ADD2|CUL3|PPFIA1|CAP2|FBXO5|CKAP2|CORO6 | -2.1 |
| GO_BP | GO:0051101 regulation of DNA binding | 3 | HDAC2|HEY1|ZBTB7A | -2.1 |
| GO_BP | GO:0042471 ear morphogenesis | 3 | ABR|SOD1|GJB6 | -2.1 |
| GO_BP | GO:0097305 response to alcohol | 4 | ADCY3|HDAC2|SOD1|SETDB1 | -2.1 |
| GO_BP | GO:0002576 platelet degranulation | 3 | ANXA5|FCER1G|SOD1 | -2.1 |
| GO_BP | GO:0045471 response to ethanol | 3 | HDAC2|SOD1|SETDB1 | -2.1 |
| GO_BP | GO:0008015 blood circulation | 6 | ABR|ATP1B1|SMAD5|SOD1|THRB|KCNMB4 | -2.1 |
| GO_BP | GO:0048872 homeostasis of number of cells | 4 | FCER1G|SMAD5|SOD1|ZBTB7A | -2.1 |
| GO_BP | GO:0071560 cellular response to transforming growth factor beta stimulus | 4 | HDAC2|SMAD5|ZFYVE9|ZBTB7A | -2.1 |
| GO_BP | GO:0016358 dendrite development | 4 | HDAC2|SHANK2|FBXW8|SLC12A5 | -2.1 |
| GO_BP | GO:0007423 sensory organ development | 6 | ABR|CALB1|HDAC2|SOD1|THRB|GJB6 | -2 |
| GO_BP | GO:0003013 circulatory system process | 6 | ABR|ATP1B1|SMAD5|SOD1|THRB|KCNMB4 | -2 |
| GO_BP | GO:0071559 response to transforming growth factor beta | 4 | HDAC2|SMAD5|ZFYVE9|ZBTB7A | -2 |
| GO_CC | GO:0000118 histone deacetylase complex | 3 | HDAC2|ZNF217|ZBTB7A | -3.1 |
| GO_CC | GO:0098984 neuron to neuron synapse | 6 | ABR|ADD2|CALB1|SYN2|CAP2|SHANK2 | -2.9 |
| GO_CC | GO:1990234 transferase complex | 8 | HDAC2|CUL3|FBXW8|HCFC2|ZBTB7A|PHC3|AMN1|CCNY | -2.5 |
| GO_CC | GO:0030424 axon | 7 | ABR|DAGLA|CALB1|SOD1|PPFIA1|SHANK2|NRSN1 | -2.3 |
| GO_CC | GO:0098794 postsynapse | 7 | ABR|ADD2|DAGLA|CALB1|SYN2|CAP2|SHANK2 | -2.3 |
| GO_CC | GO:0014069 postsynaptic density | 5 | ABR|ADD2|SYN2|CAP2|SHANK2 | -2.3 |
| GO_CC | GO:1904949 ATPase complex | 3 | ATP1B1|HDAC2|ZBTB7A | -2.3 |
| GO_CC | GO:0032279 asymmetric synapse | 5 | ABR|ADD2|SYN2|CAP2|SHANK2 | -2.3 |
| GO_CC | GO:0099572 postsynaptic specialization | 5 | ABR|ADD2|SYN2|CAP2|SHANK2 | -2.2 |
| GO_CC | GO:0033267 axon part | 5 | DAGLA|CALB1|SOD1|SHANK2|NRSN1 | -2 |
| GO_MF | GO:0001227 DNA-binding transcription repressor activity, RNA polymerase II-specific | 5 | SMAD5|ZNF217|HEY1|ZBTB7A|HMBOX1 | -2.9 |
| GO_MF | GO:0051015 actin filament binding | 4 | ADD2|GJB6|ANTXR1|CORO6 | -2.4 |
| GO_MF | GO:0003682 chromatin binding | 6 | HDAC2|THRB|SETDB1|NCAPD3|HCFC2|FANCM | -2 |
| KEGG Pathway | hsa04213 Longevity regulating pathway - multiple species | 3 | ADCY3|HDAC2|SOD1 | -3 |
| KEGG Pathway | hsa04976 Bile secretion | 3 | ADCY3|ATP1B1|NCEH1 | -2.8 |
| KEGG Pathway | hsa04911 Insulin secretion | 3 | ADCY3|ATP1B1|KCNMB4 | -2.6 |
| KEGG Pathway | hsa04919 Thyroid hormone signaling pathway | 3 | ATP1B1|HDAC2|THRB | -2.2 |

BP, biological processes; CC, cellular components; MF, molecular function; GO, gene ontology; KEGG, Kyoto encyclopedia of genes and genomes.
