# Supplementary figures and images for: Identification of potential biomarkers and candidate small molecule drugs in glioblastoma
Source: Cancer Cell Int. 2020 Aug 28;20:419. doi: 10.1186/s12935-020-01515-1 (PMC7455906; doi:10.1186/s12935-020-01515-1)

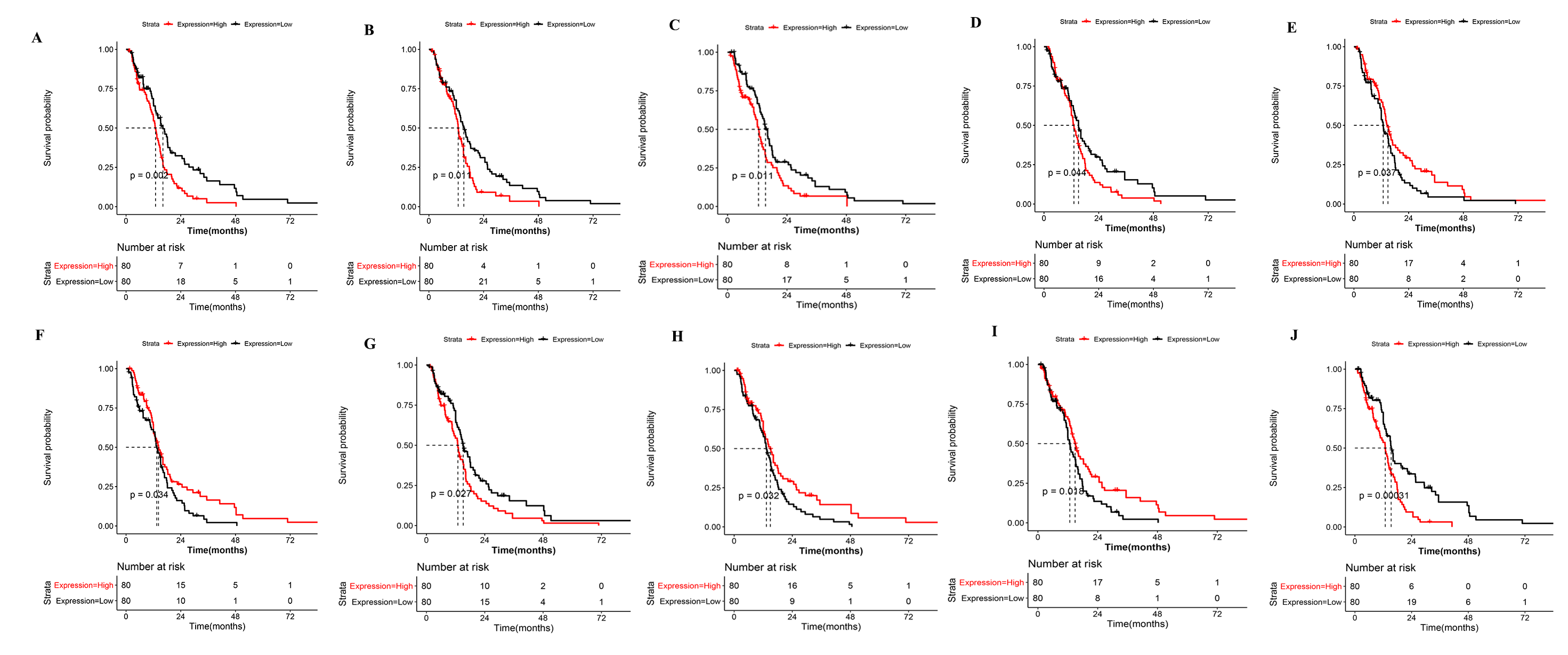

Supplement: Supplementary file 4 — Additional file 4: Fig. S1. Survival analysis for hub genes in GBM. Kaplan–Meier plots show 10 hub genes related to overall survival rate (P < 0.05). A: CETN2, B: MKI67, C: ARL13B, D: SETDB1, E: CALN1, F: ELAVL3, G: ADCY3, H: SYN2, I: SLC12A5, J: SOD1. [file 12935_2020_1515_MOESM4_ESM.tif]

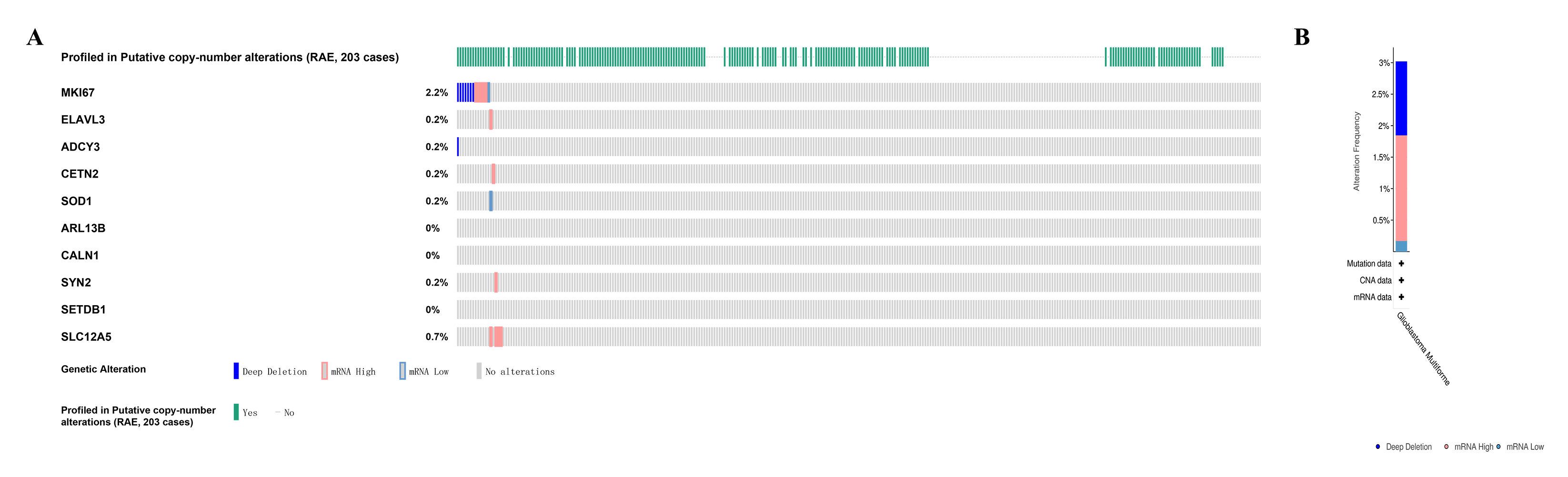

Supplement: Supplementary file 5 — Additional file 5: Fig. S2. Gene mutation frequencies of hub genes. A: The mRNA alterations of hub genes. The dark blue bars represent deep deletion, the pink bars represent mRNA up-regulation, the pool blue bars represent mRNA down-regulation, and gray bars represent no alteration. B: Percentage of gene mutations in GBM patients. [file 12935_2020_1515_MOESM5_ESM.tif]
